# Management of Clinically Solitary Papillary Thyroid Carcinoma Patients According to Risk-Scoring Model for Contralateral Occult Carcinoma

**DOI:** 10.3389/fendo.2020.553577

**Published:** 2020-10-08

**Authors:** Jia-Wei Feng, Jing Ye, Wan-Xiao Wu, Hua Pan, An-Cheng Qin, Yong Jiang, Bao-Qiang Wu

**Affiliations:** ^1^Department of Thyroid Surgery, The Third Affiliated Hospital of Soochow University, Changzhou First People’s Hospital, Changzhou, China; ^2^Department of General Surgery, The Affiliated Suzhou Hospital of Nanjing Medical University, Suzhou Municipal Hospital, Suzhou, China; ^3^Department of General Surgery, The Second People’s Hospital of Changzhou affiliated to Nanjing Medical University, Changzhou, China

**Keywords:** papillary thyroid microcarcinoma, contralateral occult carcinoma, ipsilateral occult carcinoma, recurrence-free survival, surgery

## Abstract

**Objective:**

The aim of this study was to investigate risk factors of occult carcinoma in clinically solitary papillary thyroid carcinoma (PTC) patients, and to put emphasis on the predictive value of risk-scoring model to determine the optimal scope of surgery

**Methods:**

A total of 573 clinically solitary PTC patients who underwent total thyroidectomy (TT) from two hospitals were retrospectively analyzed. Clinicopathological features were collected, univariate and multivariate analyses were performed to determine risk factors of occult carcinoma. The Cox proportional hazards model was used to analyze the risk factors of recurrence. A scoring model was constructed according to independent risk factors of contralateral occult carcinoma.

**Results:**

19.2% of clinically solitary PTC patients had occult carcinoma, among which 3.7% patients had ipsilateral occult carcinoma and 15.5% patients had contralateral occult carcinoma. Factors such as male, the presence of benign nodule, and vascular invasion increase the risk of ipsilateral occult carcinoma. Tumor size >1 cm, the presence of benign nodule, extrathyroidal extension, central lymph node metastasis, lateral lymph node metastasis are independent predictors of contralateral occult carcinoma. Contralateral occult carcinoma is the independent predictor of recurrence. A 10-point risk-scoring model was established to predict the contralateral occult carcinoma in clinically solitary PTC patients.

**Conclusion:**

Lobectomy is sufficient for clinically solitary PTC patients with risk factors of ipsilateral occult carcinoma. For clinically solitary PTC patients with score ≥4, careful preoperative evaluations are required to rule out the contralateral occult carcinoma. Even if contralateral occult carcinoma is not detected preoperatively, TT is recommended for high-risk patients.

## Introduction

Papillary thyroid carcinoma (PTC) is the most common endocrine malignant tumor worldwide, accounting for approximately 80.0% of thyroid malignant tumors (1, 2). Although the incidence of PTC has increased rapidly in incidence, understanding of PTC has become more and more thorough from the molecular, diagnostic, and prognostic perspectives. According to the 2004 World Health Organization “Thyroid and Parathyroid Diseases and Genetic Classification”, in addition to the most common classic PTC, there are 15 subtypes of PTC, among which highly aggressive PTC variants, such as diffuse sclerosing, tall-cell, insular, and poorly differentiated subtypes, exhibit heterogeneous clinical behavior and a wide range of mortality risk (3). Fine needle aspiration (FNA) of thyroid nodules can detect different gene mutations and other molecular changes in PTC, and the use of molecular pathways has permitted the development of new targeted therapies for aggressive PTC (4).

Despite the progress has been made in understanding the basic biological characteristics of PTC, the optimal scope of surgery, lobectomy vs total thyroidectomy (TT), is still controversial. As reported, PTC frequently occurs as multifocal lesions, with the prevalence of multifocality ranging from 20.0 to 36.1% (5–7). For tumors preoperatively detected in the bilateral lobes, there is no controversial to perform TT. Considering the possibility of recurrent or persistent carcinoma in the remnant contralateral lobe, TT seems to be applicable for tumors confined to the unilateral lobe. The rate of contralateral occult PTC discovered by TT was reported to range from 10 to 30% (8–11). Considering the high incidence of postoperative complications, the routine use of TT is not recommended for all patients with unilateral PTC. However, multifocal PTCs were reported to be associated with poor outcome, and increased risk of recurrence (7, 12, 13). High resolution of ultrasonography (US), which could detect foci as small as 2–3 mm, is currently used in detecting PTC and determining the scope of surgery (14). However, smaller tumors remain undetected. In addition, the ability of US to detect small malignant tumors would be significantly reduced when patients had diffuse thyroid disease (15).

Therefore, identifying risk factors of occult carcinoma in the thyroid lobe may help surgeons determine the optimal scope of surgery. By using a large series of patients who underwent initially TT for the treatment of a single PTC confined to only the unilateral lobe, we aimed to identify risk factors of occult carcinoma. In addition, we investigated the effects of occult carcinoma on clinical outcomes of patients. Different from previous studies, we established a model based on the risk factors of occult carcinoma. According to this risk-scoring model, we can predict high-risk populations of occult carcinoma among solitary PTC patients for the avoidance of unnecessary TT, and provide individualized treatment for high-risk populations of occult carcinoma.

## Materials and Methods

### Patients

This multi-center retrospective cohort study consisted of patients from Changzhou First People’s Hospital and Suzhou Municipal Hospital between January 2011 and January 2018. These patients underwent initially TT for the treatment of a single PTC confined to only the unilateral lobe, without other suspicious carcinoma lesions in the contralateral lobe by preoperative image examinations or FNA. The Institutional Review Board of Changzhou First People’s Hospital and Suzhou Municipal Hospital approved this retrospective study. All participants gave written informed consent for their clinical records to be used in this study. Patients were excluded from the study if they have any of following factors: (1) patients with preoperative clinical evidence of multiple PTCs or other pathologic types of thyroid malignancies; (2) patients with the single PTC had another suspicious lesion, but FNA was not performed for the suspicious lesion; (3) non-PTCs (medullary/follicular/anaplastic) or other subtypes than classic PTC (such as mixed PTC and so on); (4) patients did not undergo the TT; (5) patients had another malignancy before thyroidectomy; (6) patients with diffuse thyroid disease; (7) reoperation; (8) distant metastasis at diagnosis on pathological or clinical analysis; (9) history of neck radiation or familial cancer; (10) incomplete clinical data or missing follow-up. Finally, a total of 573 patients were enrolled in this study.

### Surgical Procedures

We used the US, computed tomography (CT), or FNA to evaluate primary lesions and lymph nodes in the neck preoperatively. All patients underwent TT. TT was defined as the removal of two lobes, the isthmus, and the pyramidal lobe. For patients with clinically positive central lymph node metastasis (CLNM), therapeutic central neck dissection (CND) would be performed. All clinically lymph node-negative (cN0) PTC patients underwent prophylactic central neck dissection (pCND). CND plus therapeutic ipsilateral lateral neck dissection (LND) were performed for patients with clinically positive or intraoperative suspected lateral lymph node metastasis (LLNM). The central compartment refers to level VI. CND and pCND included the removal of prelaryngeal, pretracheal, and bilateral paratracheal lymph nodes (16). LND was performed in the usual fashion from at least level II to level V, sparing the internal jugular vein, spinal accessory nerve, and sternocleidomastoid muscle (17). According to neck levels, all lymph nodes specimens were separated by the surgeon and were sent to the department of pathology for paraffin fixation and histological analysis.

### Histopathologic Examination of Surgical Specimens

Two or more experienced pathologists reviewed and cross-checked all pathology specimens microscopically. Contralateral occult carcinoma, the tumor lesion detected by pathology postoperatively in the contralateral lobe rather than detected by preoperative examinations. Similarly, ipsilateral occult carcinoma was the tumor lesion detected by pathology postoperatively in the ipsilateral lobe rather than detected by preoperative examinations. Two or more PTC foci within the thyroid was defined as multifocality. Two or more PTC foci in a single lobe were ipsilateral multifocality, while two or more PTC foci in both lobes were bilateral multifocality. Papillary thyroid microcarcinoma (PTMC) was defined as PTC ≤1 cm in its maximum diameter. Extrathyroidal extension (ETE) was defined as the primary tumor extending through the thyroid capsule to perithyroidal soft tissue, or involving strap muscles, or extending to surrounding structures (18). PTC was subdivided into the following three groups according to the position: upper pole (upper part of the high plane of the isthmus), middle pole (parallel to the isthmus), and lower pole (lower part of the low plane of the isthmus). The location of the tumor was determined by the largest dominant lesion when the patient had multifocal lesions. When the dominant lesion occupied 2 adjacent parts, the location of the tumor was determined by the portion containing more than two-thirds of the tumor volume. Recurrence was defined as the any new lesions detected in cervical lymph nodes, or other organs on cytology from aspiration biopsy. Recurrence-free survival (RFS) was used to evaluate the outcomes. RFS was the duration started from the surgery to recurrence.

### Postoperative Management and Follow-Up

Postoperative suppressive levothyroxine (LT4) treatment was conventionally performed in all patients. Thyroid-stimulating hormone (TSH) suppression therapy (serum TSH level below 0.5 mIU/L) with LT4 with or without radioactive iodine (RAI) ablation was administered to patients who underwent TT. After the initial surgery, physical examinations, US of the neck, and serum thyroid function (free thyroxin, TSH, thyroglobulin and anti-thyroglobulin antibodies) were performed for all patients every 6 months for a period of 2 years, and thereafter once a year. The criteria for remission was defined as (1) no evidence of tumor recurrence in clinical or radiologic examination, and (2) serum thyroglobulin levels of <2 ng/ml during TSH stimulation and <1 ng/ml during TSH suppression in the absence of anti-thyroglobulin antibodies. Disease recurrence, which included the local, regional, and distant recurrence, was defined as new evidence of pathologically proven recurrence in patients who initially met the criteria of remission. After the radiographic or biochemical examinations, histological examination of new lesion would be performed to verify whether the lesion was the recurrent PTC.

### Statistical Analyses

All statistical analyses were performed using the SPSS v 25.0 software (Chicago, IL, USA). The continuous variables were expressed as means ± standard deviations (SD). Univariate analysis for the comparison between patient groups was used Pearson’s chi-square test or Fisher’s exact test. Variables with a *P* <0.05 in the univariate analysis were included in the multivariate analysis, which were performed logistic regression analysis to assess risk factors of contralateral/ipsilateral occult carcinoma. The potential relationship between clinicopathological variables and recurrence was used the Cox proportional hazards model to analyze. A risk-scoring model was constructed to calculate the probability of contralateral occult carcinoma on the basis of results in the multivariate analysis. The independent risk factors were selected as scoring items. According to the beta coefficient obtained from the logistic regression model, the score of each risk factor was weighted. To make the scoring model simple, all the beta coefficient divided the least one and then rounded to the nearest whole number. The total score for each patient represented the sum of scores for each risk factor. Receiver operating characteristic (ROC) curve was used to evaluate the predictive performance of the scoring model and find an appropriate cut-off point.

## Results

### Base Clinicopathological Characteristics of Patients

The baseline clinicopathological characteristics of patients are summarized in **Table 1**. Among the 573 PTC patients, there were 134 men and 439 women with the mean age of 44.7±12.3 years (range from 19 to 80 years). The mean BMI was 22.39±5.34 kg/m^2^ (range from 11.03 to 39.06 kg/m^2^), and 208 (36.3%) patients were overweight. The diameter of the tumors ranged from 0.10 to 6.00 cm with the mean diameter of 1.20±0.87 cm. Among 64 PTC patients who performed BRAF mutation analysis, 56 (87.5%) patients had BRAF mutation positivity. Histopathological examination of specimens showed that one hundred and six (18.5%) patients had benign thyroid nodules. Occult PTC foci were detected in the contralateral lobe in 89 (15.5%) patients, and in the ipsilateral lobe in 21 (3.7%) patients. The ETE and vascular invasion were detected in 84 (14.7%) and 28 (4.9%) patients, respectively. Tumor located in the upper portion of the thyroid gland was detected in 221 (38.6%) patients, and tumor located in the middle/lower lobe of thyroid was detected in 352 (61.4%) patients. CLNM only was present in 207 patients (36.1%), both CLNM and LLNM were present in 64 patients (11.2%), and LLNM only was present in 20 patients (3.5%). The mean number of removed and metastatic lymph nodes in the central compartment was 6.0±4.6 and 2.5±1.5, respectively. And the mean number of removed and metastatic lymph nodes in the lateral compartment was 18.3±11.5 and 5.0±4.8, respectively.

**Table 1:** Clinicopathological characteristics of 573 PTC patients.

Clinicopathological characteristics	No. (%)
Sex	
Male	134 (23.4%)
Female	439 (76.6%)
Age (Y), Mean±SD (range)	44.7 ± 12.3 (19–80)
≥55	113 (19.7%)
<55	460 (80.3%)
BMI (kg/m^2^), Mean±SD (range)	22.39 ± 5.34 (11.03–39.06)
Normal	365 (63.7%)
Overweight	208 (36.3%)
Maximum tumor size (cm), Mean±SD (range)	1.20 ± 0.87 (0.10–6.00)
≤1	283 (49.4%)
>1	290 (50.6%)
BRAF mutation*	
Absence	8 (12.5%)
Presence	56 (87.5%)
With benign nodule	
Absence	467 (81.5%)
Presence	106 (18.5%)
Occult carcinoma	
Absence	463 (80.8%)
Contralateral occult carcinoma	89 (15.5%)
Ipsilateral occult carcinoma	21 (3.7%)
ETE	
Absence	489 (85.3%)
Presence	84 (14.7%)
Vascular invasion	
Absence	545 (95.1%)
Presence	28 (4.9%)
Tumor location	
Upper	221 (38.6%)
Middle/Lower	352 (61.4%)
LNM	
Without LNM	282 (49.2%)
CLNM only	207 (36.1%)
LLNM only	20 (3.5%)
CLNM and LLNM	64 (11.2%)
No. of removed LNs in CC, Mean±SD (range)	6.0 ± 4.6 (2–32)
No. of removed LNs in LC, Mean±SD (range)	18.3 ± 11.5 (5–51)
No. of metastatic LNs in CC, Mean±SD (range)	2.5 ± 1.5 (0–18)
No. of metastatic LNs in LC, Mean±SD (range)	5.0 ± 4.8 (3–22)
Recurrence	31 (5.4%)
LNs	29 (5.1%)
Lung	2 (0.3%)

PTC, papillary thyroid carcinoma; Y, year; SD, standard deviation; ETE, extrathyroidal extension; LNM, lymph node metastasis; CLNM, central lymph node metastasis; LLNM, lateral lymph node metastasis; LN, lymph node; CC, central compartment; LC, lateral compartment.

*BRAF mutation analysis was started in 2017 and it was performed in 64 patients with PTC.

### Clinicopathological Factors Associated With Contralateral Occult Carcinoma

Among 89 patients with occult lesions in the contralateral gland, 31 patients had occult lesions in the upper portion of the thyroid glands, and 58 patients had occult lesions in the middle/lower lobe poles of the glands. The mean size of the contralateral occult lesion was 0.27±0.19 cm (range 0.10–0.35 cm). No contralateral occult lesions exhibited the ETE. In **Table 2**, contralateral occult carcinoma presented the significant association with tumor size, the presence of benign nodule, ETE, vascular invasion, CLNM, and LLNM by univariate analysis (all *P* < 0.05). All of these factors were included in the multivariate analysis and showed that tumor size >1 cm (OR: 2.280, 95% CI: 1.111–4.680, *P* = 0.025), the presence of benign nodule (OR: 7.361, 95% CI: 3.678–14.731, *P* < 0.001), ETE (OR: 15.324, 95% CI: 7.428–31.615, *P* < 0.001), CLNM (OR: 4.125, 95% CI: 1.914–8.891, *P* < 0.001), LLNM (OR: 6.983, 95% CI: 3.492–13.966, *P* < 0.001) remained independent predictors of contralateral occult carcinoma.

**Table 2:** Associations between clinicopathological characteristics and contralateral occult carcinoma in PTC patients.

Variables	Contralateral occult	Solitary	*P* value	OR	95% CI	*P* value
	N = 89 (16.1%)	N = 463 (83.9%)				
Sex						
Female	22 (24.7%)	95 (20.5%)				
Male	67 (75.3%)	368 (79.5%)	0.375			
Age (Y)						
≥ 55	12 (13.5%)	95 (20.5%)				
< 55	77 (86.5%)	368 (79.5%)	0.124			
BMI (kg/m^2^)						
Normal	62 (69.7%)	290 (62.6%)				
Overweight	27 (30.3%)	173 (37.4%)	0.206			
Tumor size (cm)						
≤ 1	22 (24.7%)	253 (54.6%)		1		
> 1	67 (75.3%)	210 (45.4%)	<0.001	2.280	1.111–4.680	0.025
BRAF mutation*						
Absence	1 (7.1%)	6 (12.8%)				
Presence	13 (92.9%)	41 (87.2%)	0.919			
With benign nodule						
Absence	44 (49.4%)	409 (88.3%)		1		
Presence	45 (50.6%)	54 (11.7%)	<0.001	7.361	3.678–14.731	<0.001
ETE						
Absence	41 (46.1%)	440 (95.0%)		1		
Presence	48 (53.9%)	23 (5.0%)	<0.001	15.324	7.428–31.615	<0.001
Vascular invasion						
Absence	80 (89.9%)	459 (99.1%)		1		
Presence	9 (10.1%)	4 (0.9%)	<0.001	1.026	0.224–4.708	0.974
Tumor location						
Upper	39 (43.8%)	172 (37.1%)				
Middle/Lower	50 (56.2%)	291 (62.9%)	0.236			
CLNM						
Absence	15 (16.9%)	277 (59.8%)		1		
Presence	74 (83.1%)	186 (40.2%)	<0.001	4.125	1.914–8.891	<0.001
LLNM						
Absence	45 (50.6%)	427 (92.9%)		1		
Presence	44 (49.4%)	36 (7.8%)	<0.001	6.983	3.492–13.966	<0.001

PTC, papillary thyroid carcinoma; Y, year; BMI, body mass index; ETE, extrathyroidal extension; CLNM, central lymph node metastasis; LLNM, lateral lymph node metastasis; OR, Odds ratio; 95% CI, 95% confidence interval.

*BRAF mutation analysis was started in 2017 and it was performed in 61 PTC patients in this table.

### Clinicopathological Factors Associated With Ipsilateral Occult Carcinoma

Of 21 patients with occult lesions in the ipsilateral gland, 6 patients had occult lesions in the upper portion of the thyroid glands, and 15 patients had occult lesions in the middle/lower lobe poles of the glands. The mean size of the ipsilateral occult lesion was 0.24±0.16 cm (range 0.10–0.31 cm). No ipsilateral occult lesions exhibited the ETE. As summarized in **Table 3**, univariate analysis revealed that male, the presence of benign nodule, ETE, and vascular invasion were significantly associated with the presence of ipsilateral occult carcinoma (all *P* < 0.05). All of these factors were included in the multivariate analysis and revealed that male (OR: 45.286, 95% CI: 4.819–425.574, *P* = 0.001), the presence of benign nodule (OR: 9.858, 95% CI: 2.120–45.842, *P* = 0.004), and vascular invasion (OR: 68.081, 95% CI: 7.440–662.304, *P* < 0.001) were independent predictive factors of ipsilateral occult carcinoma.

**Table 3:** Associations between clinicopathological characteristics and ipsilateral occult carcinoma in PTC patients.

Variables	Ipsilateral occult	Solitary	*P* value	OR	95% CI	*P* value
	N = 21 (4.3%)	N = 463 (95.7%)				
Sex						
Female	4 (19.0%)	368 (79.5%)		1		
Male	17 (81.0%)	95 (20.5%)	<0.001	45.286	4.819–425.574	0.001
Age (Y)						
≥ 55	6 (28.6%)	95 (20.5%)				
< 55	15 (71.4%)	368 (79.5%)	0.539			
BMI (kg/m^2^)						
Normal	13 (61.9%)	290 (62.6%)				
Overweight	8 (38.1%)	173 (37.4%)	0.946			
Tumor size (cm)						
≤ 1	8 (38.1%)	253 (54.6%)				
> 1	13 (61.9%)	210 (45.4%)	0.137			
BRAF mutation*						
Absence	1 (33.3%)	6 (12.8%)				
Presence	2 (66.7%)	41 (87.2%)	0.378			
With benign nodule						
Absence	14 (66.7%)	409 (88.3%)		1		
Presence	7 (33.3%)	54 (11.7%)	0.010	9.858	2.120–45.842	0.004
ETE						
Absence	8 (38.1%)	440 (95.0%)		1		
Presence	13 (61.9%)	23 (5.0%)	<0.001	2.152	0.139–33.219	0.583
Vascular invasion						
Absence	6 (28.6%)	459 (99.1%)		1		
Presence	15 (71.4%)	4 (0.9%)	<0.001	68.081	7.440–662.304	<0.001
Tumor location						
Upper	10 (47.6%)	172 (37.1%)				
Middle/Lower	11 (52.4%)	291 (62.9%)	0.333			
CLNM						
Absence	10 (47.6%)	277 (59.8%)				
Presence	11 (52.4%)	186 (40.2%)	0.265			
LLNM						
Absence	17 (81.0%)	427 (92.2%)				
Presence	4 (19.0%)	36 (7.8%)	0.153			

PTC, papillary thyroid carcinoma; Y, year; BMI, body mass index; ETE, extrathyroidal extension; CLNM, central lymph node metastasis; LLNM, lateral lymph node metastasis; OR, Odds ratio; 95% CI, 95% confidence interval.

*BRAF mutation analysis was started in 2017 and it was performed in 50 PTC patients in this table.

### Predictors of RFS

Postoperative follow-up ranged from 7 to 89 months (average follow-up period: 32 months). During follow-up, 31 (5.4%) patients developed recurrent disease, including 29 (5.1%) patients had cervical lymph nodes recurrence and 2 (0.3%) patients had lung recurrence.

Cox regression model in relation to RFS was conducted to determine variables which influenced recurrence. Our results showed tumor size, bilaterality, ETE, and CLNM were factors associated with recurrence (all *P* < 0.05). Multivariate analyses showed tumor size >1 cm (HR: 2.147, 95% CI: 1.005–4.585, *P* = 0.048), bilaterality (HR: 5.818, 95% CI: 2.196–15.415, *P* < 0.001), ETE (HR: 2.447, 95% CI: 1.033–5.793, *P* = 0.042), and CLNM (HR: 5.230, 95% CI: 1.818–15.046, *P* = 0.002) were independent risk predictors of recurrence, while other investigated variables had no significant influence on RFS (**Table 4**).

**Table 4:** Cox proportional hazards model demonstrating factors associated with recurrence-free survival in PTC patients.

Variables	Univariate analyses			Multivariate analyses*		
	HR	95% CI	*P* value	HR	95% CI	*P* value
Sex						
Male	1					
Female	1.652	0.706–3.865	0.247			
Age (Y)						
< 55	1					
≥ 55	0.758	0.324–1.771	0.522			
BMI (kg/m^2^)						
Normal	1					
Overweight	1.841	0.875–3.872	0.108			
Tumor size (cm)						
≤ 1	1			1		
> 1	2.621	1.094–6.280	0.031	2.147	1.005–4.585	0.048
Multifocality						
Solitary	1			1		
Ipsilateral Multifocality	1.262	0.639–2.490	0.503	1.398	0.339–5.757	0.643
Bilaterality	2.560	1.040–6.299	0.041	5.818	2.196–15.415	<0.001
ETE						
Absence	1			1		
Presence	2.560	1.040–6.299	0.041	2.447	1.033–5.793	0.042
Vascular invasion						
Absence	1					
Presence	1.390	0.560–3.450	0.477			
Tumor location						
Middle/Lower	1					
Upper	1.322	0.659–2.650	0.432			
CLNM						
Absence	1			1		
Presence	5.047	1.712–14.877	0.003	5.230	1.818–15.046	0.002
LLNM						
Absence	1					
Presence	1.091	0.529–2.250	0.814			

PTC, papillary thyroid carcinoma; Y, year; BMI, body mass index; ETE, extrathyroidal extension; CLNM, central lymph node metastasis; LLNM, lateral lymph node metastasis; HR, Hazard ratio; 95% CI, 95% confidence interval.

*The multivariate models were performed using a backward stepwise selection procedure. All clinically relevant variables with P < 0.05 in univariate results were used in the multivariate model.

### Development of Risk-Scoring Model to Predict Contralateral Occult Carcinoma

Considering that the bilaterality was the independent risk predictor of recurrence, we established the risk-scoring model to predict the contralateral occult carcinoma in solitary PTC patients. As shown in **Table 5**, based on the beta coefficient of the five independent risk factors (tumor size, the presence of benign nodule, ETE, CLNM, and LLNM) identified in the multivariate analysis of contralateral occult carcinoma, a 10-point risk-scoring model was constructed.

**Table 5:** Development of a 10-point risk-scoring model to predict contralateral occult carcinoma in PTC patients.

Variables	*P* value	Beta coefficient	Point
Tumor size (cm)			
≤ 1			
> 1	0.025	0.824	1.000
With benign nodule			
Absence			
Presence	<0.001	1.996	2.000
ETE			
Absence			
Presence	<0.001	2.729	3.000
CLNM			
Absence			
Presence	<0.001	1.417	2.000
LLNM			
Absence			
Presence	<0.001	1.944	2.000

PTC, papillary thyroid carcinoma; CLNM, central lymph node metastasis; LLNM, lateral lymph node metastasis; ETE, extrathyroidal extension.

**Figure 1:**
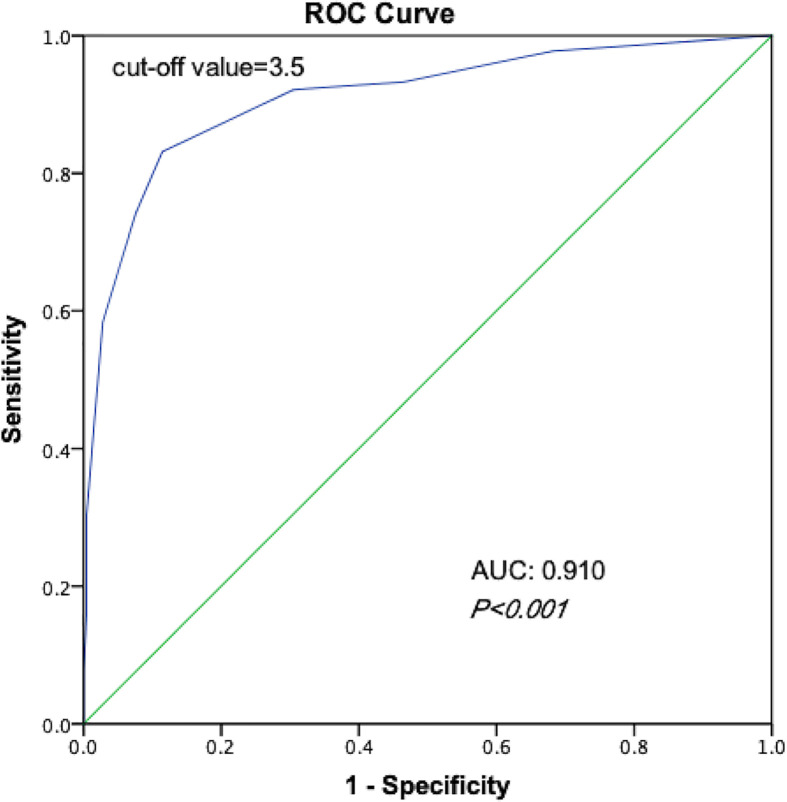
Receiver operating characteristic curves of the ability of risk-scoring model to predict the risk of contralateral occult carcinoma.

According to the scoring model, the percentage of positive contralateral occult carcinoma ranged from 1.3 to 100.0% in order of total score in PTC patients (**Table 6**). A ROC curve of the risk-scoring model for contralateral occult carcinoma was plotted, and the area under the curve of the model for the prediction of contralateral occult carcinoma was 0.910 (95% CI: 0.872–0.948, *P* < 0.001), indicating that the discriminative power of this model is acceptable (**Figure 1**). Moreover, a total score of 3.5 with the highest Youden’s J value (0.717) was selected as the appropriate cut-off value for the model. Patients with a total score between 0 and 3 had the low risk of contralateral occult carcinoma, while patients with a total score ranging from 4 to 9 had the high risk of contralateral occult carcinoma.

**Table 6:** Risk scores and percentage of contralateral occult carcinoma in PTC patients.

Risk score	contralateral occult carcinoma (+)	contralateral occult carcinoma (–)	Total	Positive rate (%)
0	2	147	149	1.3%
1	4	101	105	3.8%
2	1	74	75	1.3%
3	8	88	96	8.3%
4	8	18	26	30.8%
5	14	22	36	38.9%
6	25	11	36	69.4%
7	13	0	13	100.0%
8	7	2	9	77.8%
9	7	0	7	100.0%

PTC, papillary thyroid microcarcinoma.

## Discussion

Continued attention has been paid to PTC given its increasing incidence, which is reaching epidemic proportions. The treatment of PTC, especially PTMC, remains the controversial topic. According to the latest American Thyroid Association (ATA) guidelines, TT is recommended for patients with tumor size >4cm, gross maximal ETE (T4), cervical lymph node metastasis, or distant metastasis. For preoperatively detected bilateral PTMC patients, TT is generally accepted, while for unilateral PTMC patients without ETE or cervical lymph node metastasis, lobectomy may be sufficient. In addition, considering that PTMC behaves more like a benign lesion, some scholars have proposed less aggressive strategies, such as “watch-and-wait” rather than surgery (19). However, some PTMCs may be occult and accompanied by aggressive behavior, which may lead to the local recurrence and cervical lymph node metastasis (8). In this study, we investigated the association between clinicopathological characteristics and occult carcinoma in order to identify solitary PTC patients with high risk of occult carcinoma, who would clearly benefit from a more extensive treatment.

In our study, we used the entire thyroid to determine the prevalence and distribution pattern of occult carcinoma in 573 consecutive patients with isolated nodule detected by preoperative US. Different from previous studies which defined “occult carcinoma” as the undetected carcinoma in the contralateral lobe when TT was performed in patients with preoperative unilateral PTC (20), we divided the occult carcinoma into contralateral and ipsilateral occult carcinoma. Our study found that 19.2% of clinically solitary PTC patients had occult carcinoma, among which 3.7% patients had ipsilateral occult carcinoma and 15.5% patients had contralateral occult carcinoma. The incidence of contralateral occult cancer in this study is consistent with the 10 to 30% reported in previous studies (8–11). Considering serious complications of TT, such as vocal cord paralysis and hypoparathyroidism, the 15.5% incidence of contralateral occult carcinoma cannot convince all patients with clinically solitary PTC to routinely undergo TT. Hence, we further examined any clinicopathological characteristics related to the presence of occult carcinoma, especially contralateral occult carcinoma, to help select high-risk patients with occult carcinoma.

In this study, factors such as male, the presence of benign nodule, and vascular invasion were independent predictors of ipsilateral occult carcinoma. As for contralateral occult carcinoma, tumor size >1 cm, the presence of benign nodule, ETE, CLNM, LLNM were independent predictors. Our results were consistent with previous studies regarding the risk factors of multifocality (21–23). To our surprise, the presence of benign nodule was the risk factor of both contralateral and ipsilateral occult carcinoma. This result may be due to the presence of occult small carcinoma obscured by benign nodules. Moreover, the accurate of US in detecting lesions with diameters less than 5 mm was only 53.8% (24), and 7.9 to 18% of the aspirates were classified as “indeterminate” by FNA, which carry the risk of malignancy (25, 26). Therefore, the presence of benign nodule in the lobe by preoperative evaluation should be regarded as the significant predictive factor of potential presence of the occult carcinoma in the both contralateral and ipsilateral lobe.

Due to the excellent prognosis of PTMC, some people hold the view that the treatment of PTMC should be different from PTC (19). We further analyzed risk factors of recurrence. In addition to the tumor size >1 cm, ETE, and CLNM, which previously confirmed to be associated with recurrence (27–30), we found bilaterality, that is, the occult carcinoma in the contralateral lobe, is also the independent predictor of recurrence. However, ipsilateral multifocality, that is, the occult carcinoma in the ipsilateral lobe, has no association with recurrence. This result was consistent with previous studies. For example, a recent study involving 2,211 Chinese PTC patients showed the 10-year disease-free survival rate of patients with bilateral PTC was much lower than that of those with unilateral-multifocal and solitary PTC (78.8 vs 85.7 and 89.3%, respectively; *P* = 0.005) (31). Moreover, a study of 2,095 patients with PTC who underwent TT found that multifocality rather than bilaterality was the independent predictor of disease recurrence or persistence (32). Therefore, conservative treatment, such as lobectomy, can be performed for clinically solitary PTC patients with risk factors of ipsilateral occult carcinoma.

In addition to showing contralateral occult carcinoma risk stratification data, we developed and validated a 10-point risk-scoring model to predict the presence of contralateral occult carcinoma in clinically solitary PTC patients based on tumor size, the presence of benign nodule, ETE, CLNM, and LLNM in our study. Predictive models can not only understand the response probabilities of each predictor to other factors, but also quickly assess the response probabilities of individual subjects. According to the ROC curve, the area under the curve of the model for the prediction of contralateral occult carcinoma was 0.910 (95% CI: 0.872–0.948, *P* < 0.001), showing the high discrimination accuracy of the scoring model. The average incidence of contralateral occult carcinoma in PTC patients with score ≥4 (high risk) was significantly higher than that in patients with score ≤3 (low risk) (58.3 vs. 3.5%, *P* < 0.001). The risk scoring model is simple and effective, which could be implemented in clinical work. By identifying the high-risk groups in the relatively objective manner, it can provide the personalized treatment for these patients. For example, combined with preoperative image examinations, clinically solitary PTC patients with score ≥4 are recommended to undergo extensive treatment, such as TT, to improve the prognosis, while clinically solitary PTC patients with score ≤3 should undergo the lobectomy in consideration of high complication rates of TT

There are several potential limitations in our study, which expects to be extended upon in the further researches. First, our research was a retrospective study, which might have the selection bias. Compared with the prospective studies, retrospective studies tend to have more errors and biases. For instance, the data we provided were extracted from the document and were not captured in the actual conversation. The possibility of residual confounding variables of unmeasured factors could not be ruled out. Second, different surgeons were involved in performing TT and lymph node dissection. Postoperative results may be affected by surgeon-specific factors. Third, since LND is not generally recommended as a prophylactic procedure, there may be undetected LLNM. Moreover, the follow-up time was relatively short (average time: 32 months), which may lead to a low recurrence rate. Longer follow-up period may make the result more stable. Finally, more external validation, such as validation of the risk-scoring model in other institutions or other countries, is still necessary in the future.

In conclusion, our study found that male, the presence of benign nodule, and vascular invasion increase the risk of ipsilateral occult carcinoma in clinically solitary PTC patients. And tumor size >1 cm, the presence of benign nodule, ETE, CLNM, LLNM were independent predictors of contralateral occult carcinoma in clinically solitary PTC patients. Considering that bilaterality (occult carcinoma in the contralateral lobe) is the independent predictor of recurrence, we developed a 10-point risk-scoring model for contralateral occult carcinoma to better guide the treatment in clinically solitary PTC patients. For clinically solitary PTC patients with score ≥4, careful preoperative evaluations are required to exclude the contralateral occult carcinoma. Even if contralateral occult carcinoma is not detected preoperatively, TT is recommended for high-risk patients.

## Data Availability Statement

The raw data supporting the conclusions of this article will be made available by the authors, without undue reservation.

## Ethics Statement

This study has been approved by the Institutional Review Board of Changzhou First People’s Hospital and Suzhou Municipal Hospital ethics committee, and has been performed according to the ethical standards laid down in the 1964 Declaration of Helsinki.

## Author Contributions

A-CQ took charge of conceiving and designing the study. HP and W-XW were responsible for collecting the data and analyzing and interpreting the data. J-WF took charge of writing the manuscript. JY was responsible for providing critical revisions. B-QW participated in the revision of this manuscript and put forward important revisions. Approving the final version of the manuscript was in charge of YJ. All authors contributed to the article and approved the submitted version.

## Conflict of Interest

The authors declare that the research was conducted in the absence of any commercial or financial relationships that could be construed as a potential conflict of interest.

## Abbreviations

PTC, papillary thyroid carcinoma; TT, total thyroidectomy; US, ultrasonography; FNA, fine-needle aspiration; CT, computed tomography; CND, central neck dissection; LND, lateral neck dissection; pCND, prophylactic central neck dissection; CLNM, central lymph node metastasis; cN0, clinically lymph node-negative; PTMC, papillary thyroid microcarcinoma; ETE, extrathyroidal extension; RFS, recurrence-free survival; LT4, levothyroxine; TSH, thyroid-stimulating hormone; SD, standard deviations; ROC, receiver operating characteristic; ATA, American Thyroid Association.
